# Spin-Polarized Electron Transport Promotes the Oxygen Reduction Reaction

**DOI:** 10.1021/acsnano.5c14333

**Published:** 2025-10-14

**Authors:** Priscila Vensaus, Yunchang Liang, Jean-Philippe Ansermet, Jonas Fransson, Magalí Lingenfelder

**Affiliations:** † Max Planck-EPFL Laboratory for Molecular Nanoscience and Technology, École Polytechnique Fédérale de Lausanne (EPFL), Lausanne 1015, Switzerland; ‡ Institute of Physics (IPHYS), École Polytechnique Fédérale de Lausanne (EPFL), Lausanne 1015, Switzerland; § Instituto de Nanosistemas, Escuela de Bio Y Nanotecnologías, Universidad Nacional de San Martín, San Martín, Buenos Aires B1650, Argentina; ∥ Department of Physics and Astronomy, 8097 Uppsala University, Uppsala 75236, Sweden; ⊥ Helvetia Institute for Science and Innovation, Wollerau 8832, Switzerland

**Keywords:** oxygen reduction reaction, spin diffusion length, spin polarization, two-electron current, electrocatalysis

## Abstract

Oxygen evolution (OER) and oxygen reduction (ORR) reactions are central to the efficiency of electrolysis and fuel cells, involving the paramagnetic triplet ground state of oxygen and the singlet ground state of water. Here, we demonstrate that spin-polarized currents enhance the ORR activity. Using a silver-coated nickel electrode over a neodymium (Nd) magnet, we observed that ORR performance is maximized when the Ag layer is thinner than the spin diffusion length of silver—conditions under which spin alignment at the electrode–electrolyte interface is maintained. In contrast, experiments with thicker Ag layers lead to spin relaxation and diminished electrocatalytic activity. A model description of this system shows that a substantial spin polarization at the interface is accompanied by a large two-electron transfer, which satisfies conservation of angular momentum during ORR. These findings highlight the critical role of spin-selective charge transfer and offer insights into the control of reaction pathways in oxygen electrocatalysis.

## Graphical abstract

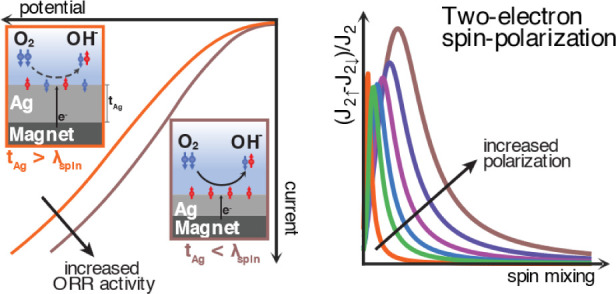

## Introduction

Oxygen electrocatalysis plays a critical role in the hydrogen economy, as it determines the efficiency of electrolysis and fuel cells.^1,2^ A comprehensive understanding of the mechanisms of the oxygen evolution (OER) and oxygen reduction (ORR) are therefore needed for efficient catalysts design. Because the oxygen molecule is paramagnetic, its production or its reactivity is intrinsically spin-dependent. The ground state of the oxygen molecule is a triplet state (↑O=O↑), while water and hydroxide are singlet states. Thus, in the transformation from O_2_ to OH^–^ (ORR, eq 1), electron transfer must be accompanied by a change in spin angular momentum.^3–9^ While overall this process encompasses either a 2 + 2 or a 4 electron pathway depending on the electrode and conditions used,^10–12^ for both cases it is unclear whether it begins with one concerted two-electron step or two sequential single electron steps.

1 $$O_{2}+4e^{-}+2H_{2}O\to 4OH^{-}$$

Increasing attention has been paid to using magnetism-related effects to enhance oxygen electrocatalysis.^13–15^ The enhancement has been assigned to the improvement of several elemental steps of the reactions caused by the magnetic field, including the reaction kinetics^8,9,16–21^ and the mass transport processes.^22–26^ Nonetheless, a complete understanding of spin effects is lacking.

Theoretical studies by Gracia and coworkers,^16,27,28^ have demonstrated that spin-dependent properties extend beyond electron transfer to include orbital interactions between catalysts and reactants or intermediates. Two main factors appear to be at play: (i) the magnetic properties of the catalyst: exchange interactions of reactants or intermediates with ferromagnetic surfaces affect their binding energies, leading to enhanced catalytic efficiencies. (ii) both OER and ORR involve the transfer of four electrons, where spin alignment is needed for the formation or consumption of the triplet oxygen molecule. Thus, spin-selective channels are needed to direct the appropriate electron spins toward the catalytic interface. Recent evidence indicates that the electrons involved in ORR are transferred in two pairs.^29–31^

Instead of using magnetic electrodes to transfer electrons with polarized spins, the chiral induced spin-selectivity (CISS) effect can be used. The CISS effect refers to the phenomenon where electron transport through chiral molecules becomes spin-dependent, leading to a preferential transmission of electrons with a specific orientation.^32–34^ Recent studies have highlighted the significant impact of this effect on both the OER and ORR activity.^6,7,35^ For instance, Naaman and coworkers established that electron transfer efficiency is enhanced in chiral electrodes during ORR.^36^

Researchers have also shown that chiral molecules adsorbed on nonferromagnetic substrates can induce magnetization, suggesting that hydrodynamic contributions may contribute to the activity enhancements reported in CISS-based ORR measurements. This highlights the importance of disentangling hydrodynamic influences from intrinsic spin effects in both magnetic and CISS-enhanced oxygen electrocatalysis. Here, we carefully distinguish these effects by keeping the magnetohydrodynamic effects constant across all samples, and we demonstrate that the transfer of spin-polarized electrons increases the catalytic activity of a material that has a nonmagnetic surface.

## Results and Discussion

Our motivation was to test the effect of spin alignment in a nonferromagnetic material, as it could explain the oxygen electrocatalysis enhancements observed in CISS electrodes. To explore this possibility, we utilized a Ni-covered Nd magnet^37^ as the working electrode (WE), coated with Ag layers of varying thickness (Figure 1a). The Nd magnet provides a strong, static magnetic field. The electrical current from the potentiostat flows into the surrounding conductive, ferromagnetic Ni layer. A spin-polarized current is thus injected from the Ni layer into the Ag overlayer, which serves as the spin transport and catalytic medium. Ag was selected due to its known activity for the ORR^10,38^ and its relatively long spin diffusion length.^39–42^ This approach allows for the direct investigation of electron spin effects on catalysis, which are distinct from the magnetohydrodynamic phenomena that would be induced by placing a conventional nonmagnetic electrode in an external magnetic field.^26^

**Figure 1:**
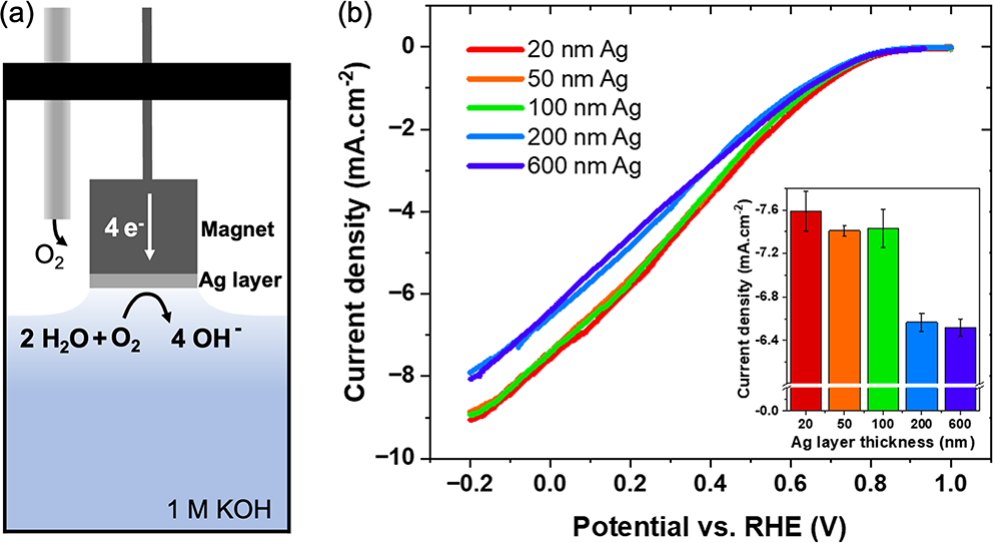
ORR activity. a) Measurement schematics: a Ni-covered Nd magnet, covered with an Ag layer, is used as the WE in a hanging-meniscus configuration in O_2_-saturated 1 M KOH, while O_2_ is continuously purged inside the electrochemical cell. b) Anodic wave in the kinetic- and mixed-controlled region of the CV. Inset shows the current density at 0 V (w.r.t. RHE) vs increasing Ag layer thickness, and error bars refer to the values taken from the last three stabilized CV cycles.

Another ingredient that is key to the interpretation of current enhancements is that most catalysts reconstruct under reaction conditions, expressing changes in the surface area and the chemical composition exposed at the electrode/electrolyte interface under reaction conditions.^43^ To account for morphological changes and remove other influences like differences in magnetic properties or binding energies, we changed only the thickness of the Ag layer, and related all ORR activities to the electrochemically active surface area (ECSA), obtained by Pb underpotential deposition (UPD) for each sample (Figure S1 and Table S1).^35,44^ Additionally, representative STM images show the samples have similar electronic contrast and topographical surface features (Figure S2).

In the charge-transfer limited region of the ORR polarization curves (Figure 1b), we observed that the current has a maximum value when the Ag layer thickness is below 100 nm, while for thicker layers the current decreases.

Since the room-temperature Ag spin diffusion length (λ_spin_) is approximately 130–150 nm in polycrystalline films,^39^ it is the spin polarization of electrons reaching the electrode that changes when the Ag thickness (*t*_Ag_) is less or more than 100 nm. When the Ag layer is thinner than 100 nm, the electrons are spin-polarized; they are not when the Ag layer is much thicker (Figure 2).

**Figure 2:**
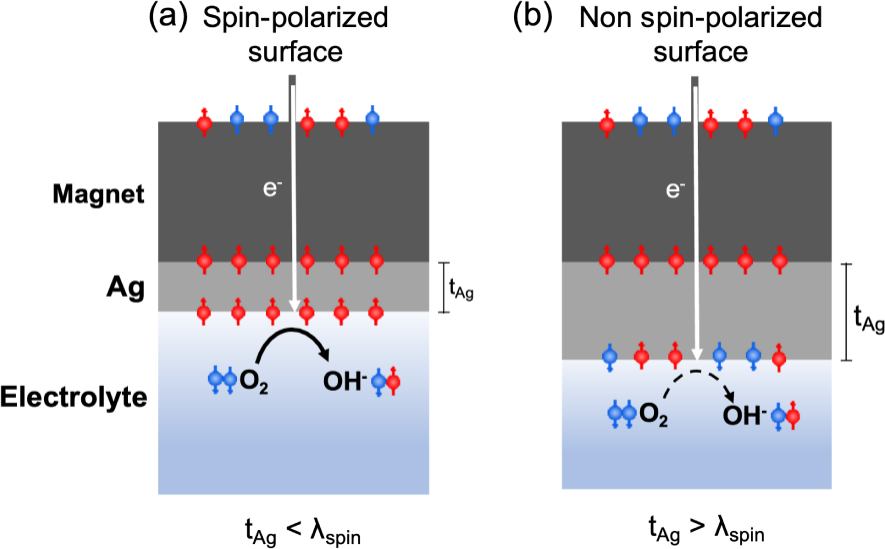
Schematics of the electron transfer process. If the Ag layer thickness is smaller than the spin diffusion length (*t*_Ag_ < λ_spin_), the electrons reaching the surface are strongly spin-polarized, and the ORR is enhanced (a); the ORR is less likely if the layer is thicker (b).

This observation is consistent with the process of spin relaxation within the Ag layer. In a nonmagnetic metal such as silver, the spin–orbit coupling creates a finite probability for an electron’s spin to flip during any momentum-scattering event (e.g., with phonons or impurities). Therefore, as electrons traverse a thicker film, they undergo more scattering events, progressively losing their initial spin polarization.^45–47^ When the Ag layer thickness exceeds the spin diffusion length, a majority of the electrons reaching the electrode–electrolyte interface are no longer spin-polarized, which explains the diminished ORR enhancement.

To confirm that the observed enhancement originates from the magnet, a control experiment was performed using an Ag layer on a Ni foil without the magnet; this sample showed significantly lower ORR activity (Figure S3). The permanent magnet has two effects on the system: (1) it saturates the magnetization of the Ni layer, spin-polarizing the transmitted electrons, and (2) it induces a Lorentz force in the electrolyte that enhances mass transport.^26^ Our experimental design, which varies the thickness of the nonmagnetic Ag layer, is specifically intended to decouple these two effects. While spin relaxation depends on the layer thickness, the contribution from the Lorentz force is expected to be constant across all samples. This is because the variation in Ag thickness is negligible compared to the dimensions of the macroscopic magnet (5 mm). Magnetostatic calculations (see Supporting Information) confirm that the magnetic field strength at the electrode surface, and thus the resulting Lorentz force, varies by less than 0.1% across this range. Such small difference cannot account for the large variations observed in ORR activity.

Additional control experiments using the achiral spin-independent one-electron redox couple [Ru­(NH_3_)_6_]^2+/3+^ revealed no significant differences in peak positions or currents with varying Ag layer thickness (Figure S4), supporting that the observed ORR dependence arises from spin-polarization effects rather than from changes in the general electrochemical properties of the electrodes.

An analogous observation was recently made using the CISS effect by Liang et al. for the OER,^35^ and by Naaman and coworkers for the ORR.^36^ In the latter, the authors showed that spin polarization obtained by chiral molecule functionalization of metal electrodes and nanoparticles leads to an enhancement of the ORR. This effect was attributed to a reduction in activation energy by allowing the reactions to happen on a triplet potential energy surface.

The underlying hypothesis of our study is that the ORR is initiated by the transfer of a concerted pair of two electrons to O_2_ and that this specific process is enhanced when the transferred electrons are spin-polarized. We emphasize that our hypothesis focuses on the nature of this initial oxygen activation step, rather than the overall electron number (n) of the complete reaction pathway. It is well-known that the single-electron current *J*_1_ is limited by the spin-polarization of the injected current, since a large portion of the electrons are uncorrelated. The two-electron current, by contrast, comprises only correlated electrons which would allow for an enhancement of the spin-polarization. We show this property of the two-electron current using a theoretical model.

We performed calculations based on a simple model description comprising the salient features of the experimental setup. Here, the active component of the oxygen molecules are two electrons which are coupled in order to sustain a spin triplet ground state. These two electrons are modeled as two spins, *s*_*m*_, *m* = 1,2, isotropically coupled in the spin triplet state. The energies that characterize the spin-dimer are the degenerate single electron levels *ε*_m_, an inter site tunneling *t*, on-site and intersite Coulomb interactions, *U* and *U*′, respectively, and a spin exchange *J*. The resulting energy landscape defines a triplet two-electron state with likely transitions to the four-electron state which is key to the ORR process.^31,48^ Details regarding the formalism for the calculations can be found in refs. 31, 48 and 49.

The spin-dimer is assumed attached to the metallic substrate that allows for an electron flux into the O_2_ molecule. The coupling Γ between the metal and the spin-dimer introduces a finite spin lifetime and enables transitions between the two- and four-particle states. In order to include the spin-diffusion in the Ag layer, a spin-mixing between the levels in the spin-dimer is added to the description.

In this calculation, we are particularly interested in the two-electron current since this is expected to provide the O_2_ molecules with electron pairs, as argued in Gupta et al.^50^ As the two-electron current can be partitioned into components in the two-electron basis {|↑,↑⟩,|↑,↓⟩,|↓,↑⟩,|↓,↓⟩,|↑↓,0⟩,|0,↑↓⟩}, we analyze the resulting currents corresponding to these projections. Here, the notation |σ,σ′⟩ = |σ⟩_1_ ⊗ |σ′⟩_2_ refers to the two-electron state with one electron per oxygen atom with spin σ and σ′, respectively, whereas the notation |↑↓,0⟩ = |↑↓⟩_1_ ⊗ |0⟩_2_ and |0,↑↓⟩ = |0⟩_1_ ⊗ |↑↓⟩_2_ refer to the two-electron states where the two electrons reside in either of the oxygen atoms, leaving the second empty.

We further allow for the possibility that the two-electron states may be mixed due to spin-mixing which originates from the spin–orbit coupling in the Ag substrate. In Figure 3 we plot the charge currents *J*_αβ_, αβ = ↑↑,↑↓,↓↑,↓↓,↑↓0,0↑↓, attributed to the two-electron processes in which two electrons are simultaneously transferred from the metal to the dioxygen, involving transitions between states of the types |↑↓,↑↓⟩⟨↑,↑| and |↑↓,↑↓⟩⟨↓,↓| where two electrons both with spin ↓ and ↑, respectively, have been transferred to the molecule. The other types of transitions can be written analogously.

**Figure 3:**
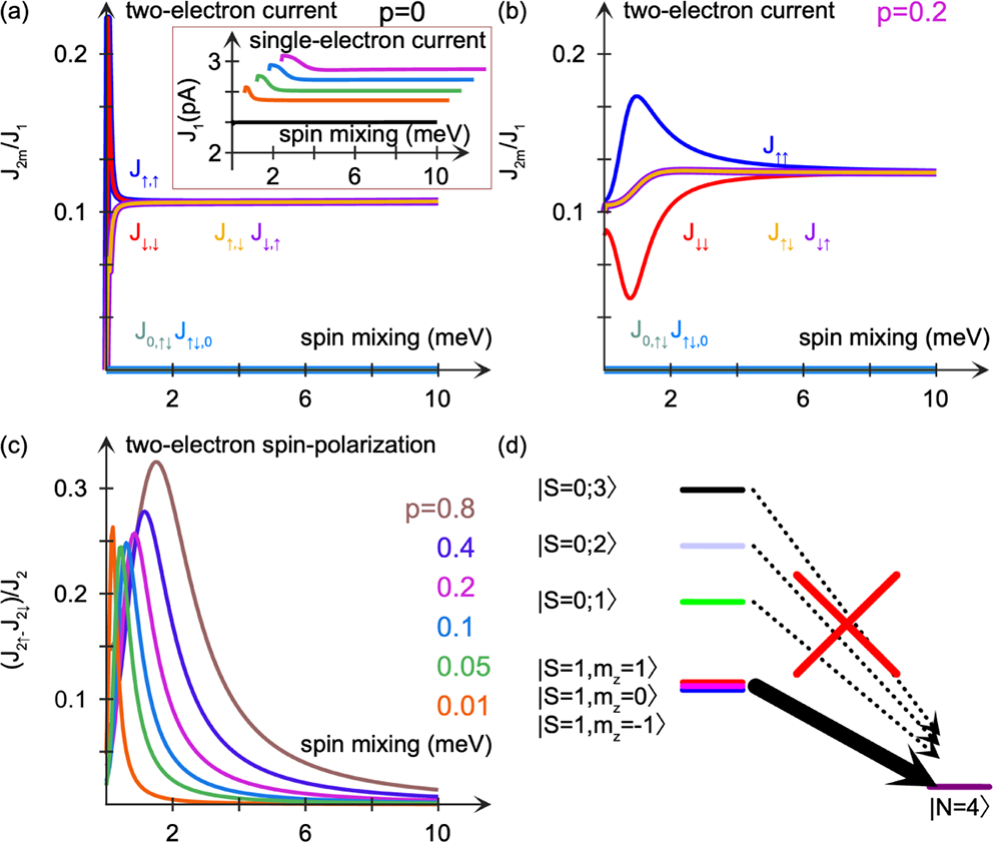
(a,b) Two-electron currents as a function of the spin-mixing resolved in the basis {|↑,↑⟩,|↑,↓⟩,|↓,↑⟩,|↓,↓⟩,|↑↓,0⟩,|0,↑↓⟩} for increasing spin-polarization *p* = *pẑ* of the injected electrons. The symbol *J*_2m_ on the vertical axis in panel (a) refers to the various two-electron currents displayed in the figure. The plotted currents are normalized by the single-electron current, which is shown in the inset in (a). (c) Spin-polarization of the two-electron current as a function of the spin-mixing for increasing spin-polarization *p*. The spin-polarization is normalized by the total two-electron current. Here, the spin-dimer is configured in terms of ε_m_ = −4 eV, *m* = 1, 2, *U* = 2 eV, *U′* = *U/*4, *J* = −(*U* – *U′*)/2, *t* = 0.1 eV, Γ_0_ = 10 meV, and temperature *T* = 300 K. (d) Schematic of the electron transfer from the spin-polarized surface to the oxygen molecule. The singlet (S = 0) states are (|↑,↓⟩ – | ↓,↑⟩)/√2, |↑↓,0⟩, and |0,↑↓⟩.

Injection of a nonspin-polarized current, Figure 3a, naturally leads to that *J*_↑↑_ = *J*_↓↓_ and *J*_↑↓_ = *J*_↑↓_, as expected, since no spin-polarization is generated in the electron transfer. Finally, the currents *J*_↑↓0_ and *J*_0↑↓_ are associated with the transitions |↑↓,↑↓⟩⟨↑↓,0| and |↑↓,↑↓⟩⟨0,↑↓|, respectively, describing the transfer of an electron pair with one spin ↑ and one spin ↓ electron. These two currents vanish, which is expected since the probability for the molecule to be in either of the states |↑↓,0⟩ or |0,↑↓⟩ is negligible.

By allowing for a spin-polarized current injection (Figure 3b,c) the two-electron currents *J*_↑↑_ and *J*_↓↓_ acquire different values for vanishing and small spin-mixing. A very weakly spin-polarized injected current leads to a substantial spin-polarization (Figure 3c). For an injected current which is spin-polarized by a mere 1%, the two-electron current spin-polarization *J*_↑↑_ – *J*_↓↓_acquires values as large as 25% of the total two-electron current *J*_2_. It should be remarked that the corresponding amplification of the spin-polarization for a single-electron current has, to our knowledge, never been reported. Any increase of the spin-polarization of the injected current beyond 1% leads only to a weak increase of the spin-polarization of the two-electron current. However, it must be stressed that this two-electron spin-polarization becomes increasingly more stable against spin-mixing when the spin-polarization of the injected current is increasingly larger. Therefore, it is important that the Ag layer is small compared to the spin diffusion length.

Thus, the model supports the experimental observation that spin correlation is crucial for enhancing ORR activity, while it does not enhance reactions involving uncorrelated single electron transfer in achiral systems. In the experiments, the control of the spin mixing was done by changing the layer thickness. We must note that the spin-dimer model is a simplified representation of the complex ORR process, which qualitatively explains spin correlation in the initial electron transfer, though it does not quantify full interfacial kinetics or account for complexities like the electric double layer or solvation. Moreover, while the model does not capture the subsequent dissipation of spin angular momentum required to complete the full reaction pathway, this can be reasonably attributed to spin relaxation mechanisms involving the substrate via spin–orbit coupling.^16,36^ Incorporating these additional factors into a more comprehensive theoretical framework remains an important avenue for future research.

## Conclusions

Summarizing, we designed a layered electrode structure that allowed us to examine the impact of spin-polarized currents on ORR, decoupled from other electrolyte or surface chemistry effects. This allowed us to demonstrate that by bringing electron spins that are polarized on the electrode surface, these spins can react with the triplet oxygen in a way that helps to conserve the angular momentum, and thus enhancing the ORR. In a broader view, this finding seems consistent with the fact that OER catalysts are materials with particular magnetic properties, such as NiFe or CoFe oxides in alkaline media, or comprise either 4–5th transition row metals like Ir or Ru, in which the rapid spin flip rate associated with high spin–orbit coupling allows for an efficient charge transfer, suggesting that electron transfer with a change in spin of the reactants could have a critical role in the reaction kinetics.^51–53^ Similar spin-related enhancements have also been reported for other catalyst systems, including Fe–N–C materials,^54,55^ Pt based alloys (Pt_3_Co),^27^ and magnetic oxides,^9^ where intrinsic spin states and spin-selective pathways influence activity. Our findings support a unifying principle for spin-enhanced catalysis: the critical requirement is not that the catalyst material itself be magnetic, but that a net spin polarization can be induced at the active site. For catalysts with intrinsically magnetic centers, such as iron sites in Fe–N–C or ferromagnetic oxides, this polarization can be accomplished directly by “spin state engineering” or external magnetic fields, respectively. Alternatively, for nonmagnetic catalysts like the silver used in our study, the active sites can be polarized indirectly by a spin-polarized current injected from an adjacent ferromagnetic layer. This work demonstrates the efficacy of this latter approach, confirming that the catalytic enhancement is not limited to materials with magnetic centers. These results also align with previous studies involving the CISS effect.

Our results provide strong evidence that supports the incorporation of spin polarizers as a way to enhance chemical reactions with spin triplet reagents. This strategy can be combined with existing approaches involving active-site optimization.^6,56^ Thus, this approach could lead to the development of more efficient catalytic systems for energy conversion and storage applications.

## Methods

All reagents were available commercially and used without further purification. Milli-Q water was used for all solution preparations. A Ni-covered NdFeB magnet (Supermagnete S-05–05-N^37^) with a magnetic field on the surface of ∼320 mT was used as substrate. Silver films were deposited at room temperature using sputtering (Balzers MED 010) from a pure silver target. The deposition occurred under controlled conditions: an argon working pressure of 0.05 mbar and a constant current of 60 mA, resulting in a deposition rate of approximately 3 Å/s. Ag thickness was controlled by the time the sample was exposed to the plasma.

For ORR measurements, on samples with 20–200 nm Ag layers, a Biologic VSP-300 potentiostat with a three-electrode cell was used. The Ag-modified magnet was used as working electrode in the hanging-drop configuration (see Figure 1a). The setup included a HydroFlex standard hydrogen reference electrode (Gaskatel) and a Pt mesh counter electrode, in 1 M KOH as electrolyte. The electrolyte was saturated with oxygen prior to each measurement, and oxygen was continuously introduced into the electrochemical cell headspace during the measurements. ORR activity was measured in static hanging-drop configuration by cyclic voltammetry until stabilization at 50 mV s^–1^, with *iR* compensation, as done in previous works.^57,58^ The sample with 600 nm Ag layer was measured in the same cell configuration, using Autolab potentiostat, a Ag/AgCl reference electrode (BaSi) and a Pt mesh counter electrode, in 1 M KOH as electrolyte. Potential was converted to RHE by *E*(vs RHE) = *E*(vs Ag/AgCl) + *E*° Ag/AgCl+ 0.059 × pH. All ORR polarization curves were taken from CVs recorded in cyclic voltammetry advanced (CVA) mode, in which the potential was fixed at −0.2 V vs RHE for 5 s before the anodic scan. In general, all CVA experiments stabilized quickly, e.g., after less than 5 cycles (Figure S5). The anodic polarization curves were corrected by the results produced with the same experimental protocol in N_2_ saturated environment. Electrochemically active surface areas were determined from Pb underpotential deposition in 0.02 M HClO_4_ and 0.002 M PbCO_3_.^35^

Control experiments with a redox couple were performed in three-electrode cell and CHI-760E potentiostat, using the Ag-modified magnet as the working electrode in the hanging-drop configuration, with a Pt counter-electrode and Ag/AgCl reference electrode. Cyclic voltammetries were performed at 100 mV/s in a solution containing 2 mM Ru­(NH_3_)_6_Cl_3_ and 0.1 M KNO_3_.

## Supplementary Material



## Notes

Pb UPD curves, electrochemical results, ECSA values, STM images and roughness analysis, magnet effects on ORR: ORR curve comparisons of the Ag layer without a magnet, magnet/spin polarization effects on an achiral one-electron reaction, non-normalized ORR polarization curves and stabilization, and magnetostatic calculations (PDF)

## #

Laboratory of Nanoscience for Energy Technologies (LNET), STI, École Polytechnique Fédérale de Lausanne, 1015 Lausanne, Switzerland
