# Supplementary material for: Spin-Polarized Electron Transport Promotes the Oxygen Reduction Reaction
Source: ACS Nano. 2025 Oct 14;19(44):38709–15. doi: 10.1021/acsnano.5c14333 (PMC12613840; doi:10.1021/acsnano.5c14333)
Supplement: Supplementary file 1 [file nn5c14333_si_001.pdf]

Spin-Polarized Electron Transport Promotes the Oxygen Reduction Reaction

Priscila Vensaus[1,2,3], Yunchang Liang[1,2], Jean-Philippe Ansermet[2], Jonas Fransson[4], Magalí Lingenfelder*[1,2,5]

1) Max Planck-EPFL Laboratory for Molecular Nanoscience and Technology, École Polytechnique Fédérale de Lausanne (EPFL), 1015 Lausanne, Switzerland
2) Institute of Physics (IPHYS), École Polytechnique Fédérale de Lausanne (EPFL), 1015 Lausanne, Switzerland.
3) Instituto de Nanosistemas, Escuela de Bio y Nanotecnologías, Universidad Nacional de San Martín, San Martín, B1650 Buenos Aires, Argentina
4) Department of Physics and Astronomy, Uppsala University, Uppsala 75236, Sweden
5) Helvetia Institute for Science and Innovation, 8832 Wollerau, Switzerland

Supplementary information

Supplementary Figures

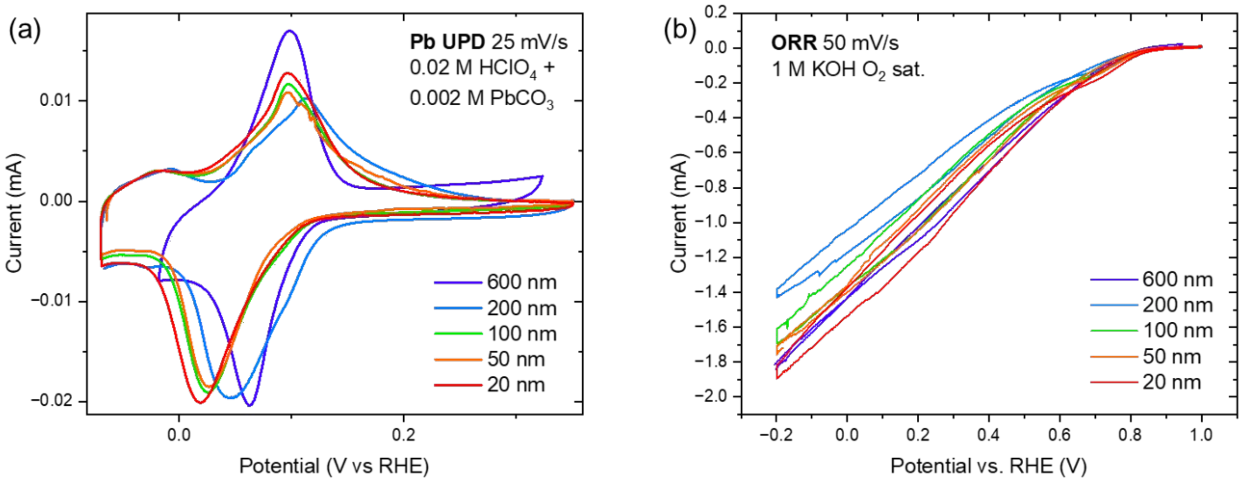

Figure S1. Electrochemical results. **a)** CVs for UPD formation of Pb/Ag on samples with different Ag layer thickness. The area of the anodic peak was used to estimate the ECSA, considering that the charge required to form a monolayer in the UPD region is 0.26 mC cm^{-2}.[44] **b)** CVs in ORR region without normalization.

Ag layer thickness (nm)	ECSA (cm^2)
20	0.201
50	0.188
100	0.183
200	0.178
600	0.217

Table S1. ECSA values obtained by Pb UPD for each sample.

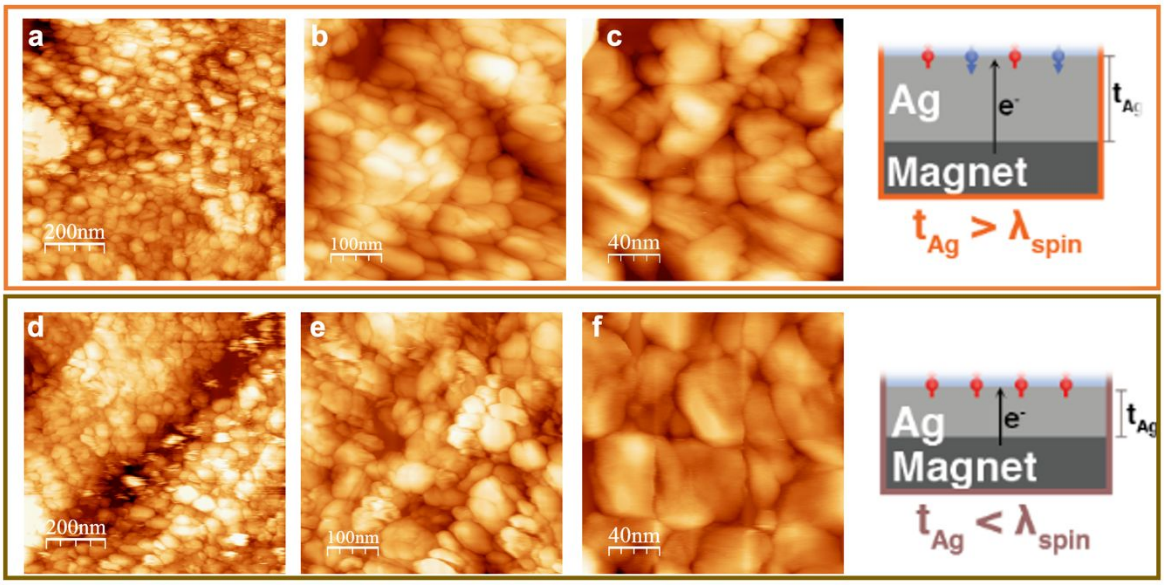

Figure S2. Representative STM images of thick Ag films on Ni/Nd magnets: a-c) 200 nm (upper panel) and d-f) 50 nm (lower panel) taken by the same tip at Bias= 0.5 mV and I=1nA, in ambient conditions. Analysis of several spots in the sample shows grains with lateral sizes in the range of ~20–60 nm. Electronic roughness RMS values for both samples were 5.0 ± 0.5 nm (taken on drift corrected images of 1 μm²), 3.5 ± 1.0 nm (on drift corrected images of 500 nm²), and 1.7 ± 0.4 nm (on drift corrected images of 200 nm²), reflecting a consistent decrease in apparent roughness with decreasing scan area. This trend is consistent with the expected increased lateral averaging and inclusion of larger topographic features at larger scan sizes. This surface characterization supports the fact that the surface of the catalyst remains similar for 50 and 200 nm thick Ag films, being the spin diffusion length the main parameter changing across samples.

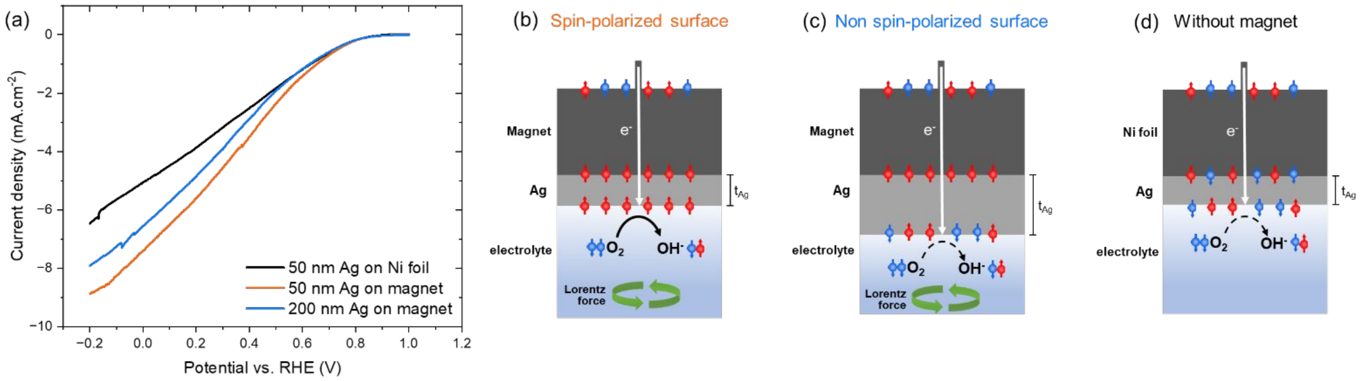

Figure S3. Magnet effects on ORR. **a)** A Ni foil coated with 50 nm Ag was used as a blank for comparison, where the electron current was not polarized due to the absence of the magnet, resulting in significantly lower current. Here, however, it is important to highlight that we can observe a lower ORR current caused by the absence of spin-polarized current along with the absence of the mass transfer enhancement caused by the magnetic field. **b-d)** Schematic of the charge transfer process. When a thin Ag layer is deposited on a magnet (b), two effects lead to an increase in the ORR current: the spin-polarization and a stirring effect generated by the Lorentz force which improves the transport of O_2 towards the electrode. If the layer is thicker (c), the spin-polarization effect is absent but the Lorentz force is still present. Without the magnet (d), none of the two effects are present and thus the ORR current is lower.

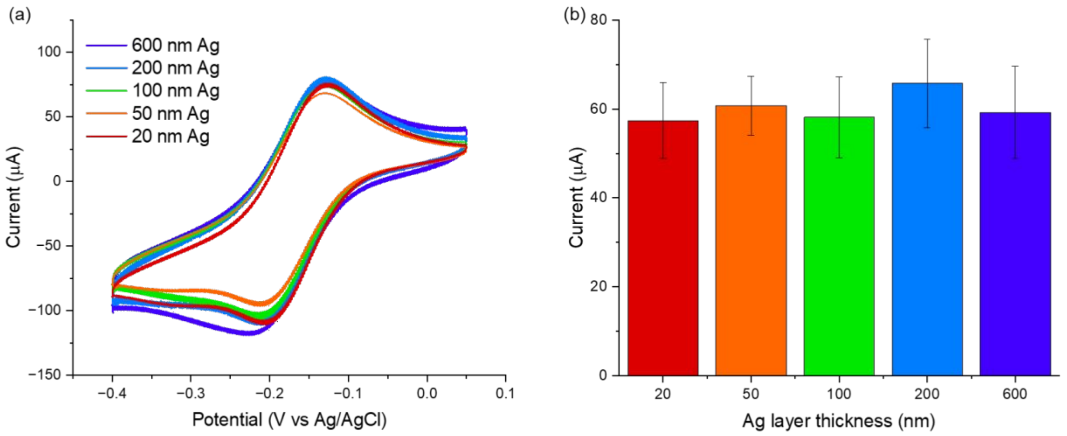

Figure S4. Magnet effects on an achiral one-electron reaction. No significant difference is observed in the peak position or peak currents. **a)** Representative CVs of Ag-coated magnets in 2 mM $Ru(NH_3)_6Cl_3$ and 0.1 M KNO_3. **b)** Anodic peak current (at ca. -0.126 V vs. Ag/AgCl) for each Ag layer thickness. Error bars show the standard deviation of multiple measurements across two independent samples for each thickness (total n = 4-6).

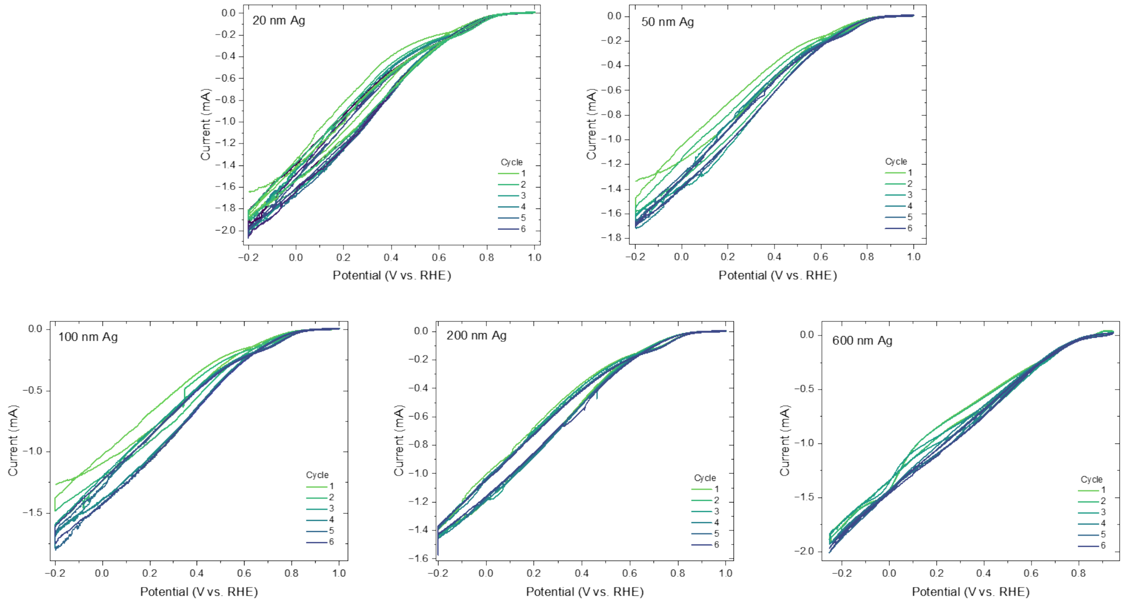

Figure S5. ORR polarization curves. CVA in O_2 saturated environment, showing stabilization after a few cycles.

Supplementary Note 1

We have performed a calculation based on the analytical model for the magnetic field on the axis of a cylindrical magnet. This calculation shows that increasing the Ag layer thickness from 20 nm to 600 nm results in a decrease in the magnetic field strength at the electrode surface of less than 0.1%.

We model the magnetic field on the central axis of the disc-shaped Neodymium magnet. Radius (R) = 2.5 mm; Thickness (D) = 5 mm. The total distance from the magnet's surface to the active interface is the sum of the nickel layer thickness (t_{Ni}) and the silver layer thickness (t_{Ag}). While we don't have an exact value for the Ni plating, we use an estimate for a typical plating, t_{Ni} = 1 µm. The Ag layer thickness varies from $t_{Ag,min}$ = 20 nm to $t_{Ag,max}$ = 600 nm.

The standard formula for the magnetic field strength on the axis of a cylindrical permanent magnet is:

$$B(z) = \frac{B_r}{2 \cdot \mu_0}\left(\frac{D+z}{\sqrt{(R^2+(D+z)^2}} - \frac{z}{\sqrt{R^2+z^2}}\right)$$

Where:
B(z) is the magnetic field strength at a distance z from the surface.
B_r is the remanence of the magnet material (a constant, typically ~1.2-1.4 T for Nd magnets).
D is the thickness of the magnet.
R is the radius of the magnet.
z is the distance from the magnet's surface.
µ0 is the vacuum magnetic permeability.

We will calculate B(z) for the two extreme cases ($t_{Ag,min}$ and $t_{Ag,max}$), where $z = t_{Ni}+t_{Ag}$. Note that B_r is a constant and will cancel out in the relative comparison. So, we have:

$$B(z_{min}) = \frac{B_r}{2 \cdot \mu_0} \cdot 0.8942$$

$$B(z_{max}) = \frac{B_r}{2 \cdot \mu_0} \cdot 0.8939$$

The percentage change is then calculated as:

$$\%\text{change} = \frac{|B(z_{min}) - B(z_{max})|}{B(z_{min})} \cdot 100\% = 0.03\,\%$$
